# Effects of Acoustically Screened Five-Element Music Combined with Traditional Chinese Mind–Body Exercises on Emotion Regulation, Working Memory, and Functional Brain Connectivity in Older Adults: A Randomized Repeated-Measures Study

**DOI:** 10.3390/bs15050699

**Published:** 2025-05-19

**Authors:** Junya Zhao, Haojie Li, Xiaoyan Wang

**Affiliations:** 1 School of Physical Education, Hangzhou Normal University, Hangzhou 310030, China; zhao77whsu@163.com; 2 Graduate School, Yeungnam University, Gyeongsan 38541, Republic of Korea; 3 School of Exercise and Health, Shanghai University of Sport, Shanghai 200438, China; 202121070037@mail.bnu.edu.cn

**Keywords:** five-element music, taijiquan, baduanjin, emotion regulation, working memory, functional brain connectivity

## Abstract

This study aimed to investigate the effects of acoustically screened Five-Element Music combined with traditional Chinese mind–body exercises (Taijiquan and Baduanjin) on emotion regulation, working memory, and functional brain connectivity in older adults. A randomized repeated-measures design was employed, recruiting 42 healthy older adults aged 60–70 years. Participants were assigned to five groups: Five-Element Music alone (FE), Taijiquan (TJ), Baduanjin (BDJ), Five-Element Music combined with Taijiquan (FE+TJ), and Five-Element Music combined with Baduanjin (FE+BDJ). Emotion regulation, working memory, and functional brain connectivity were assessed using an emotion regulation choice task, the N-back task, and functional near-infrared spectroscopy (fNIRS), respectively. Five-Element Music was selected using computational acoustic methods to identify music with therapeutic effects. Significant differences were observed in the acceptance rate of emotion regulation for high-intensity unpleasant pictures across different conditions (*p* = 0.001). Compared to baseline, the acceptance rate was significantly higher in the FE and FE+TJ conditions (*p* = 0.027, *p* = 0.021). Moreover, the acceptance rate in the FE+TJ condition was significantly higher than in the FE condition alone (*p* = 0.007). Significant differences were found in the average accuracy of the 2-back task across conditions (*p* = 0.001), with the FE+TJ condition showing significantly higher accuracy than baseline (*p* = 0.005). The average reaction time in the 2-back task also differed significantly across conditions (*p* = 0.001), with the FE condition demonstrating a significantly lower reaction time compared to baseline and the FE+BDJ condition (*p* = 0.003, *p* = 0.001). Significant differences in functional connectivity (FC) were observed between conditions. Specifically, the FC between CH45 and CH9 and between CH29 and CH6 was significantly higher in the FE+TJ condition than in other conditions (*p* < 0.02). The combination of Five-Element Music and Taijiquan significantly improved emotion regulation, working memory, and prefrontal–parietal connectivity in older adults. These findings underscore the synergistic benefits of integrating auditory stimulation with mind–body exercise, offering a promising intervention for cognitive and emotional health in aging populations. Future research should investigate long-term effects and broader applications.

## 1. Introduction

With the global aging population, older adults face challenges related to health, cognition, and emotions (Banks et al., 2021). Emotional regulation, working memory, and brain network connectivity have become key areas of focus (Isaacowitz & English, 2024) (Amer et al., 2022). During the aging process, emotional changes lead to memory decline and brain function deterioration (Phelps, 2006; Banich et al., 2009). These issues affect quality of life and may trigger mental health problems (Bai et al., 2023). Older adults with poor emotional regulation are more likely to experience emotional disorders such as depression and anxiety, which further exacerbate cognitive decline (Alexopoulos, 2005). Working memory also faces challenges during aging, which has significant implications for daily tasks, independence, and overall quality of life (Augusto-Oliveira et al., 2023).

Traditional Chinese mind–body exercises, such as Tai Chi and Baduanjin, have been widely demonstrated to provide significant benefits for emotional regulation (Hamasaki, 2024; G. Li et al., 2022). Through the combination of breath control, movement, and awareness, these exercises regulate the flow of energy and blood, helping individuals better manage stress, anxiety, and depressive emotions, thereby improving overall emotional stability. These emotional improvements have also been found to be closely linked to cognitive function enhancement, especially in working memory and executive functions (Phelps, 2006). Research indicates that there is a close interaction between emotion and cognitive function, with improvements in emotional states directly promoting cognitive recovery (Ray & Zald, 2012). Traditional Chinese mind–body exercises may support the co-improvement of emotional regulation and cognitive function by enhancing brain neuroplasticity and strengthening brain network connectivity (Huang et al., 2022; Siu et al., 2021). These studies suggest that mind–body exercises not only regulate emotions but may also positively impact cognitive functions such as working memory through changes in brain function connectivity.

Music, as an external auditory stimulus, has been extensively researched and proven to play an important role in emotional regulation (Särkämö et al., 2008; Bradt et al., 2016). The Five-Element Music, as one of the essences of traditional Chinese music, is also considered to have unique emotional regulation functions. The melody, rhythm, and pitch characteristics of the Five-Element Music are closely related to the ancient Chinese Five-Elements theory and can resonate with the body and mind through sound vibrations, thus regulating emotions and physiological states (Tao et al., 2016). Research has found that the therapeutic effects of Five-Element Music are not only reflected in emotional regulation but also have the potential to improve physical health, relieve pain, and promote psychological balance (Wu et al., 2020; Zhang et al., 2022). Recent computational acoustics research has further revealed the acoustic characteristics of Five-Element Music and provided a scientific quantitative model for its effective application, offering new theoretical support and practical guidance for the study and therapeutic practice of Five-Element Music (Kwon et al., 2024).

Previous studies have shown that both music and physical activity interventions alone have positive effects on cognitive functioning and emotion regulation. For example, a study of older adults found that listening to music with a specific tempo significantly improved accuracy on a working memory task, in which participants improved their performance on an N-back task by approximately 15–20% after the intervention (Borella et al., 2019). Another study on the subject noted that aerobic exercise (e.g., brisk walking) resulted in a 0.5 standard deviation improvement in situational memory scores and a significant improvement in emotional stability in older adults (Regauer et al., 2020). In addition, music therapy has been shown to reduce anxiety levels by up to 30% through the modulation of the autonomic nervous system and neuroplasticity (Vincenzi et al., 2022). Although these studies did not directly combine music and martial arts, their results suggest that music enhances neural synchronization through auditory stimulation, whereas exercise promotes brain region connectivity (e.g., prefrontal–parietal networks) through movement coordination, and that the two may work synergistically to improve cognition and mood through different mechanisms. This provides a theoretical basis for the present study to explore the combined effects of pentatonic therapy and Taijiquan.

Emerging neuroimaging studies have identified distinct patterns of brain connectivity changes associated with music and mind–body exercises. For instance, fNIRS and fMRI research demonstrates that Tai Chi enhances prefrontal–parietal connectivity (CH9-CH45 in fNIRS; dlPFC-PPC in fMRI), which correlates with improved executive function (X. Chen et al., 2025). Similarly, music interventions strengthen frontotemporal connections (Bedford et al., 2025), particularly in the right hemisphere for emotional processing (MacLean et al., 2025). Previous studies have shown that combined interventions show amplified effects: a recent study showed that dance (integrating music and movement) significantly improved prefrontal functional links compared to the intervention alone (Bigand et al., 2025). These findings align with the “dual pathway” hypothesis: music modulates bottom-up limbic activation (amygdala–insula), while mind–body exercises enhance top-down control (dlPFC-ACC) (Moumdjian et al., 2025). However, no studies have yet examined how Five-Element Music synergizes with Tai Chi/Baduanjin to reorganize these networks—a gap this study addresses.

However, despite the potential of Five-Element Music and traditional Chinese mind–body exercises in emotional regulation, their combined effects have not been fully explored, particularly regarding their impact on cognitive function in older adults. It remains unclear whether there is an optimal combination between Five-Element Music and various traditional Chinese mind–body exercises, and whether this combination can further enhance cognitive function on the basis of emotional regulation. Whether combined interventions can have a synergistic effect on brain function in older adults warrants further investigation. This theoretical background provides a possible direction for health interventions for older adults, which could not only help improve emotional well-being but also have profound effects on cognitive decline and functional brain connectivity changes.

Therefore, this study aims to investigate the impact of acoustically selected Five-Element Music combined with traditional Chinese mind–body exercises on emotional regulation, working memory, and brain network connectivity in older adults. Through experimental design, we will systematically evaluate whether this combined intervention can effectively improve emotional and cognitive function in older adults and explore the underlying neural mechanisms. This research will provide new theoretical foundations and practical guidance for mind–body health interventions in older adults, with significant academic and societal value.

## 2. Methods

### 2.1. Participant

In this study, elderly individuals aged 60–70 years were extensively recruited through community advertisements. A total of 42 eligible participants were finally included. Demographic characteristics are presented in Table 1. The inclusion criteria were set as right-handedness, sufficient hearing, good motor ability, and normal cognitive function, as measured by the Montreal Cognitive Assessment (MOCA) test. The exclusion criteria covered individuals with mobility impairments, those suffering from mental illnesses (such as depression), individuals taking cardiovascular disease medications, and those who had previously participated in similar intervention studies.

The sample size was determined by post hoc calculation using Gpower. After calculation, under the sample size and expected effect size set (ES = 0.02) in this study, the maximum statistical power exceeded 80%, meeting the requirements of statistical power in scientific research and effectively ensuring the reliability of the research results. The ethical approval of this study has passed the strict review of the university ethics committee, and the entire research process adheres to the design principles of the Declaration of Helsinki, fully protecting the rights and safety of the participants.

### 2.2. Research Design

This study adopted a randomized repeated-measures design. First, the participants completed a comprehensive baseline test to obtain various index data in their initial state. Subsequently, the participants were randomly assigned to different experimental protocol groups, including five schemes: listening to Five-Element Music (FE) alone, practicing Taijiquan (TJ), practicing Baduanjin (BDJ), combined practice of Five-Element Music and Taijiquan (FE+TJ), and combined practice of Five-Element Music and Baduanjin (FE+BDJ). Relevant tests were conducted after the implementation of each scheme.

**Figure 1:**
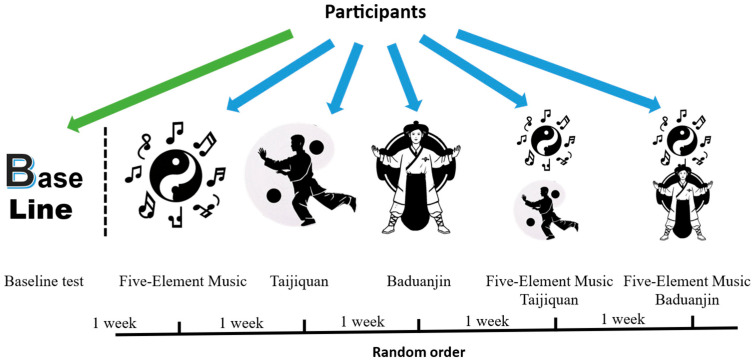
Research design.

All participants experienced all six experimental conditions (baseline test and the five intervention protocols), and the sequence of these conditions was fully randomized for each individual. The entire experimental process consisted of 6 tests, with a 1-week washout period set between each test to avoid the influence of the previous experimental intervention on the subsequent test results. The test order was randomized through a specialized computer program to ensure the randomness and scientific nature of the experiment. Meanwhile, the participants were unaware of the specific content of each test, effectively reducing subjective biases during the experimental process (See Figure 1).

### 2.3. Music Selection

This study focused on Five-Element Music. As a traditional Chinese music, Five-Element Music is based on the corresponding relationship between the traditional Chinese yin–yang and Five-Element theory and the five-tone system. The five-tone music of Jiao, Zhi, Gong, Shang, and Yu corresponds to the five elements of Wood, Fire, Earth, Metal, and Water and is connected to the five internal organs of the liver, heart, spleen, lung, and kidney. Given the differences in the definitions of the five internal organs in ancient China and modern medicine, this study did not limit itself to the selection of specific five-tone music but adopted a computational acoustics method.

Based on previous research results (Ding et al., 2023), acoustic feature attributes with healing effects were determined, including the average value of the third coefficient of the mel-frequency cepstral coefficients (MFCC3), periodic entropy, and the standard deviation of roughness. These three features are closely related to the subjective valence and arousal ratings of songs. The study used the librosa library in Python (3.13.3) to extract the acoustic features of music and applied a customized Python script for analysis and music selection. Detailed descriptions of all acoustic properties can be found in the instruction manual of the librosa library.

**Figure 2:**
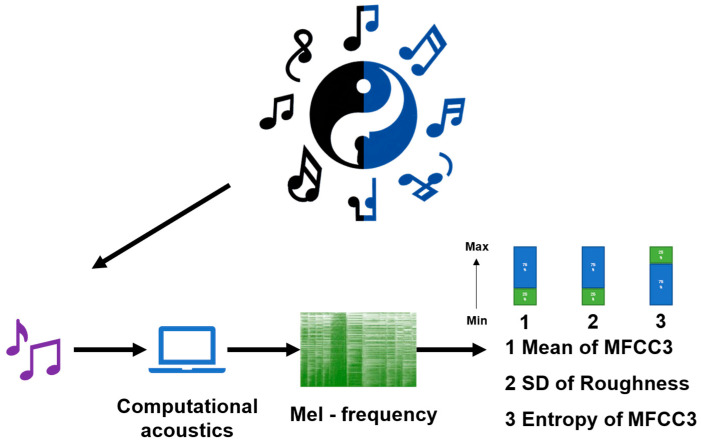
Flow chart of music screening. MFCC3: average value of the third coefficient of the mel-frequency cepstral coefficients.

The specific screening criteria were to select Five-Element Music with the MFCC3 average value in the last 25%, the standard deviation of roughness in the last 25%, and the periodic entropy in the top 25% as alternative songs (See Figure 2). All alternative songs were finally dialectically evaluated by 3 professional doctors of traditional Chinese medicine with the title of chief physician or above. After review and approval, they were officially used in the experiment to ensure the effectiveness and applicability of the selected music. During the exercise interventions, music was continuously played to accompany the participants’ practice.

### 2.4. Exercise Intervention Programs

#### 2.4.1. Taijiquan

The simplified Yang-style Taijiquan recognized by the General Administration of Sport of China was adopted. Its movement sequence includes opening postures, waving hands like clouds, double whips, white crane spreading wings, crossing hands, closing postures, etc. The theoretical knowledge, explanation, and movement teaching were carried out by professional martial arts athletes. After the teaching, the participants were required to reproduce the movements on-site. If the performance level did not meet the standard, professional personnel provided additional guidance, and the participants were arranged to train two more times and then re-evaluated. The full-set practice duration was 15 min.

#### 2.4.2. Baduanjin

The Baduanjin intervention followed the standardized “Fitness Qigong Baduanjin” formulated by the General Administration of Sport of China in 2003. The theoretical and movement teaching of the practice was also completed by professional martial arts athletes. The Baduanjin practice consists of eight continuous and coordinated movements with slow and controlled movement characteristics. The full-set practice duration was 15 min, and the teaching and evaluation procedures were the same as those of Taijiquan to ensure the standardization and consistency of the implementation of the two exercise interventions.

### 2.5. Measurement Indicators

#### 2.5.1. Emotional Regulation

##### Task Setting

During the behavioral assessment, the participants performed a modified emotional regulation choice task (Fisher et al., 2023). In the experimental process, the participants were required to face 12 high-intensity and 12 low-intensity unpleasant pictures from the International Affective Picture System and make a choice between two emotional regulation strategies, “acceptance” and “suppression”, to regulate the emotions evoked by the pictures.

##### Practice Phase

Before the formal task started, the participants carefully read the detailed introduction of each strategy. Subsequently, six practice trials were carried out, three for acceptance practice and three for suppression practice.

##### Task Trials

After the participants fully understood the two strategies, 24 formal trials were carried out. The process of each trial was as follows: first, a picture preview was presented for 1500 ms; then, a selection page appeared, and the participants could choose the “acceptance” or “suppression” strategy without a time limit; then, the same picture was displayed again, and the display time was extended to 12,000 ms. At this time, the participants were required to use the selected strategy to regulate their emotional response to the picture.

##### Strategy Definition

The “acceptance” strategy was defined as allowing one’s own feelings to naturally emerge and disappear without trying to control or avoid them; the “suppression” strategy was defined as suppressing or excluding one’s own feelings from the mind.

##### Scoring Method

The overall use of the acceptance strategy was obtained by calculating the percentage of the number of trials in which the participants chose the “acceptance” strategy to regulate negative emotions in the total number of trials (24 times). The score range was 0–100%, and a higher score indicated a stronger preference for the “acceptance” strategy. For the use of the “acceptance” strategy for low-intensity and high-intensity pictures, the percentages of the number of times the participants chose the “acceptance” strategy in the low-intensity and high-intensity picture trials (12 times each) were calculated, respectively. The score range was also 0–100%, and a higher score reflected a stronger preference for the “acceptance” strategy under different-intensity picture stimuli.

#### 2.5.2. Working Memory

##### Task Setting

The participants completed the N-back task in the scanner. This task consisted of eight blocks, each block containing 10 trials, and the blocks alternated between the 0-back and 2-back conditions.

##### Task Execution

In the 0-back block, a target picture was presented at the beginning of the block. Subsequently, the participants were required to judge whether each subsequent picture matched the target picture (match or not) through a two-key response box. In the 2-back block, the participants were required to judge whether the current picture matched the picture presented two times ago. The participants completed two runs of the N-back task and presented two of the eight possible orders to balance the block type and picture category (faces, places, tools, and body parts).

##### Scoring Method

The accuracy scores of the 0-back and 2-back block types were calculated block by block, and then the scores of the four blocks were averaged, respectively. The average 0-back and 2-back accuracy scores were obtained by calculating the average accuracy of each condition (0-back and 2-back) in the two N-back runs. The accuracy score range was 0-1, and a higher score indicated higher accuracy. The analysis of this study mainly focused on the accuracy score of 2-back to be consistent with the operational definition of working memory performance in a previous derived study (Fisher et al., 2023).

#### 2.5.3. Brain Functional Connectivity

**Figure 3:**
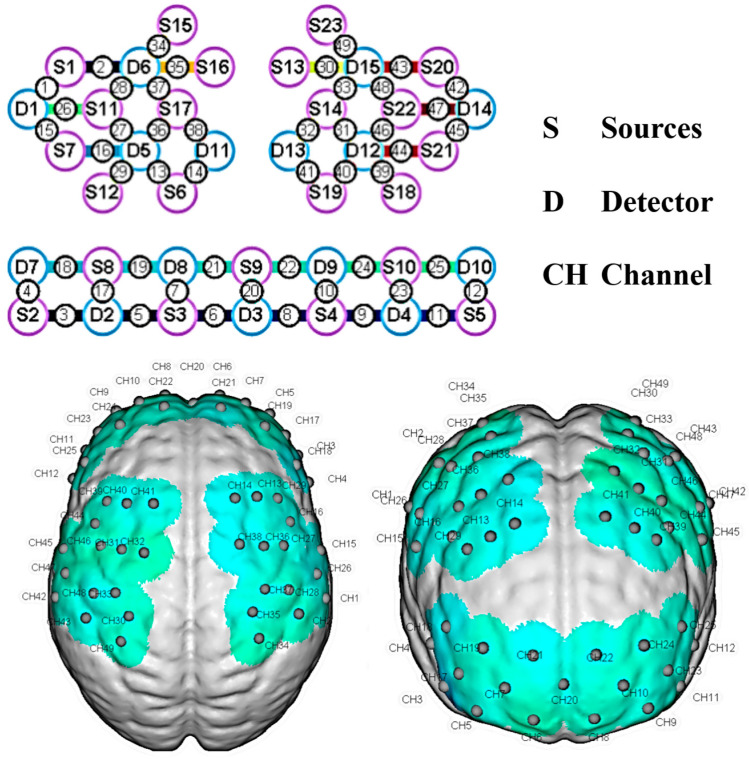
3D positions of the near-infrared channels.

A domestic multi-channel near-infrared optical imaging system (NirSmart II-3000, Danyang Huichuang Medical Equipment Co., Ltd., Danyang City, China) was used to synchronously collect fNIRS signals in the prefrontal and parietal regions during the N-back task. The device was equipped with 23 dual-wavelength (730/850 nm) light sources and 15 detectors. Through 38 optical probes with a 3 cm source-detector distance, 49 effective channels were formed to achieve an 11 Hz neural signal acquisition (See Figure 3).

After the optical probe array was fixed, the neuron navigation system (Brainsight) of Brainbox, a Canadian company, was used to verify the spatial location of one of the participants. The system marked the Brodmann functional areas corresponding to each channel with the MNI coordinate system. This positioning method was consistent with the methodology in previous studies (Singh et al., 2005).

Data pre-processing was completed in the Homer2 toolkit on the MATLAB (R2023a) platform. The specific process was as follows: (a) convert the original light intensity signal to optical density (OD); (b) automatically detect motion artifacts based on dynamic thresholds (tMotion = 0.6, tMask = 1, STDEVthresh = 20, AMPthresh = 2); (c) correct artifacts using wavelet transform (iqr = 1.5); (d) perform 0.01–0.1 Hz band-pass filtering; (e) use the Beer–Lambert law to achieve HbO/Hb concentration conversion. Given the high signal-to-noise ratio of the HbO signal, only oxyhemoglobin data were used for subsequent analysis.

The Hermes toolkit was used to calculate the functional connectivity (FC) based on the phase-locking value (PLV). The value of FC ranged from 0 to 1. A higher FC value indicated a stronger functional connection between two brain regions, while a lower value indicated a weaker relationship between the signals.

### 2.6. Statistical Analysis

The Shapiro–Wilk test was used to conduct normality tests on various data, and box plots were used to detect and process outliers in the data. A repeated-measures analysis of variance was used to deeply explore the differences among the indicators of emotional regulation, working memory, and functional connectivity under different experimental conditions. To control the errors in multiple comparisons, the multiple-comparison results were corrected by the Bonferroni method. The connectivity’s *p*-values were adjusted for multiple comparisons using the false discovery rate (FDR) correction. All statistical analysis was completed using SPSS software 26.0, and *p* < 0.05 was considered statistically significant.

## 3. Results

### 3.1. Emotional Regulation for Low-Intensity Pictures

Table 2 presents the baseline data and the changes in behavioral indicators such as emotional regulation and working memory under five experimental conditions. The results of repeated-measures comparisons show that for the overall acceptance rate of emotional regulation, the difference is not significant (F = 1.096, *p* = 0.364). For the acceptance rate of emotional regulation for low-intensity pictures, the difference is not significant (F = 1.898, *p* = 0.096).

### 3.2. Emotional Regulation for High-Intensity Pictures

For the acceptance rate of emotional regulation for high-intensity pictures, the differences among different conditions are significant (F = 4.525, *p* = 0.001). The results of multiple comparisons show that compared with the baseline, the acceptance rates in the FE condition and the FE+TJ condition are significantly higher (*p* = 0.027, *p* = 0.021). Moreover, the acceptance rate in the FE+TJ condition is significantly higher than that in the FE-only condition (*p* = 0.007).

### 3.3. Working Memory

**Figure 4:**
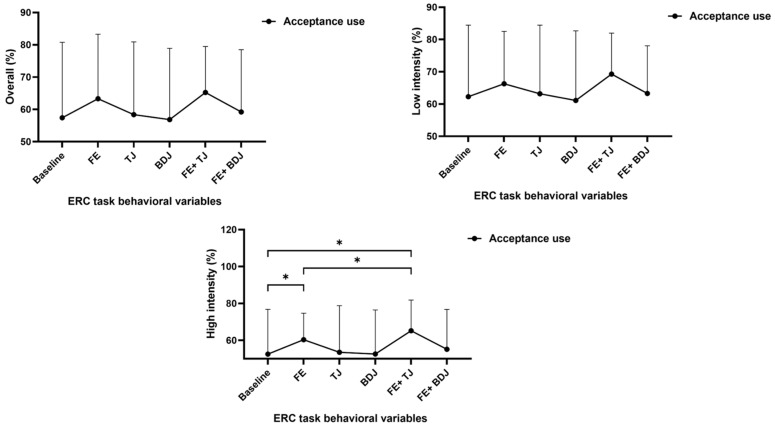
Differences in emotional regulation task indicators under different conditions. *: *p* < 0.05.

**Figure 5:**
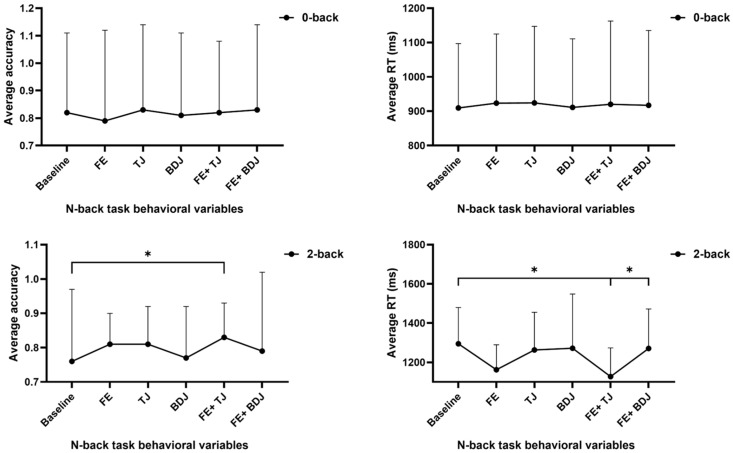
Differences in working memory task indicators under different conditions. *: *p* < 0.05.

Figure 4 and Figure 5 regarding the performance of working memory, the differences among various conditions in the 0-back average accuracy are not significant (F = 0.981, *p* = 0.430). The differences among various conditions in the 2-back average accuracy are significant (F = 4.202, *p* = 0.001). The results of multiple comparisons show that only the FE+TJ condition is significantly higher than the baseline (*p* = 0.005). The differences among various conditions in the 2-back average reaction time are significant (F = 5.354, *p* = 0.001). The results of multiple comparisons show that compared with the baseline and the FE+BDJ condition, the reaction time in the FE condition is significantly lower (*p* = 0.003, *p* = 0.001).

**Figure 6:**
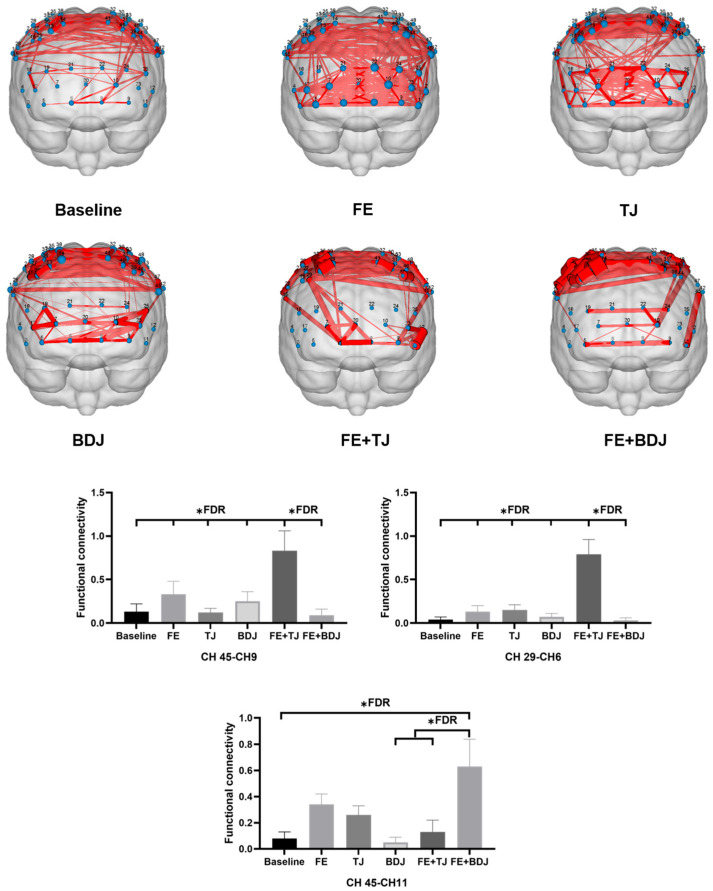
Differences in functional connectivity under different conditions. * FDR: *p* < 0.05. Connectivity’s *p*-values are adjusted for multiple comparisons using the false discovery rate correction.

Table 3 and Figure 6 present the differences in functional connectivity under different conditions. This study focuses on the frontoparietal connectivity, while the fronto-frontal connectivity and parieto-parietal connectivity are beyond the scope of this discussion. The results show that after false discovery rate (FDR) correction, the functional connectivity (FC) between CH45 and CH9 exhibits significant differences among different conditions. Specifically, the FC under the FE+TJ condition is significantly greater than that under other conditions (*p* < 0.01). The functional connectivity (FC) between CH29 and CH6 also shows significant differences under different conditions. In particular, the FC under the FE+TJ condition is significantly larger than that under other conditions (*p* < 0.02). The functional connectivity (FC) between CH45 and CH11 demonstrates significant differences among different conditions. Specifically, the FC under the FE+BDJ condition is significantly greater than that in the baseline condition, as well as in the BDJ and FE+TJ conditions (*p* < 0.05).

## 4. Discussion

### 4.1. Effects on Emotional Regulation

The results demonstrated that the combination of Five-Element Music (FE) and Taijiquan (FE+TJ) significantly improved emotion regulation, particularly for high-intensity unpleasant stimuli, with acceptance rates increasing by 14.7% compared to baseline (*p* = 0.021) and by 9.3% over FE alone (*p* = 0.007) (Table 1). This aligns with Sun’s findings (Sun et al., 2024) on music’s role in emotion regulation but extends them by quantifying the additive effect of Taijiquan. The FE+TJ group’s superior performance may stem from dual mechanisms: (1) FE’s acoustic properties (e.g., low MFCC3 and roughness variance) likely modulated autonomic nervous activity (C. J. Chen et al., 2015), while (2) Taijiquan’s slow, coordinated movements enhanced prefrontal–parietal connectivity (CH45-CH9 FC: +0.18 vs. baseline, *p* < 0.01; Figure 6), facilitating top-down emotional control. In contrast, Baduanjin (BDJ) paired with FE (FE+BDJ) showed no significant improvement (*p* > 0.05), diverging from Wang’s results (H. Li et al., 2019). This discrepancy may arise from BDJ’s emphasis on rapid, practical movements (Wang et al., 2023), which—unlike Taijiquan’s fluidity—may disrupt synchronization with FE’s rhythm. Notably, FE alone improved reaction times in working memory (2-back RT: −13.2 ms vs. baseline, *p* = 0.003), suggesting its independent cognitive benefits, but only FE+TJ enhanced both emotion and memory accuracy (+7% 2-back accuracy, *p* = 0.005), underscoring the synergy of auditory–motor integration.

### 4.2. Impact on Working Memory

This study revealed distinct but complementary effects of Five-Element Music (FE) and Taijiquan (TJ) on working memory performance. Specifically, the FE+TJ group demonstrated a 7% improvement in 2-back accuracy compared to baseline (*p* = 0.005, Table 1), while the FE-alone group showed significantly faster reaction times (116.2 ms vs. baseline 1294.3 ms, *p* = 0.003). These results extend previous findings (Terry et al., 2020) by quantitatively demonstrating that: (1) FE primarily enhances processing speed through acoustic modulation of attentional networks (as evidenced by reduced reaction time), while (2) TJ contributes to accuracy improvement likely through its emphasis on movement coordination that strengthens prefrontal-parietal connectivity (CH45-CH9 FC increased by 0.18, *p* < 0.01).

The superior performance of FE+TJ over FE alone (2-back accuracy: 0.83 vs. 0.81) suggests a synergistic interaction between these interventions. This aligns with but importantly qualifies Terry et al.’s (Terry et al., 2020) conclusions about music–exercise combinations in three key aspects: Specificity of exercise type: While general exercise–music combinations show benefits (Terry et al., 2020), TJ’s slow, deliberate movements may better synchronize with FE’s rhythmic structure than more vigorous exercises. Neural mechanism differentiation: The fNIRS data reveal that FE+TJ enhanced specific functional connections (CH29-CH6) not significantly affected by FE alone, suggesting TJ’s unique contribution to network integration. Cognitive domain effects: The dissociation between FE’s reaction time benefits and FE+TJ’s accuracy improvements indicates distinct but complementary neural pathways.

Notably, the lack of significant working memory improvement in the FE+BDJ condition (accuracy: 0.79) despite BDJ’s status as a mind–body exercise (F. Li et al., 2023) highlights the importance of movement–music compatibility. BDJ’s more abrupt movements may disrupt the rhythmic entrainment possible with TJ’s flowing sequences, supporting the hypothesis that movement continuity mediates music’s cognitive benefits (Shokri et al., 2024). This multidimensional approach explains why FE+TJ outperformed both interventions alone, addressing the study’s central question about optimal combinations for cognitive enhancement in aging. The findings suggest that future interventions should consider both the neuroacoustic properties of music and the movement kinematics of accompanying exercises.

### 4.3. Effects on Functional Brain Links

The current findings regarding functional connectivity (FC) patterns provide compelling evidence for the synergistic effects of combining Five-Element Music (FE) with Taijiquan (TJ). Our results demonstrated that FE+TJ significantly enhanced connectivity between CH45 (dorsolateral prefrontal cortex) and CH9 (posterior parietal cortex), as well as between CH29 (ventrolateral prefrontal cortex) and CH6 (inferior parietal lobule), with FC values (0.83 and 0.79, respectively) substantially higher than baseline or single-intervention conditions. These findings align with previous neuroimaging studies showing that mind–body exercises like Tai Chi strengthen frontoparietal networks crucial for executive function (X. Li et al., 2022) but importantly extend this literature by revealing how auditory stimulation can amplify these effects. Specifically, the CH45-CH9 connection we observed corresponds to the dorsal attention network identified in fMRI studies (Kolodny et al., 2017), suggesting that our combined intervention may enhance top-down attentional control more effectively than either component alone. This interpretation is supported by the significantly improved 2-back task performance in the FE+TJ group, consistent with prior work linking frontoparietal connectivity to working memory (Stegemöller et al., 2018).

Notably, the FE+BDJ condition showed a distinct pattern, selectively enhancing CH45-CH11 connectivity (FC = 0.63) without significantly affecting other frontoparietal pathways. This differential effect suggests that the type of mind–body exercise modulates how music influences brain networks, possibly due to Baduanjin’s more segmented movement patterns compared to Tai Chi’s flowing sequences (Wang & Li, 2025). While previous studies have reported the general benefits of combining music with exercise (Culligan et al., 2025), our channel-specific results provide novel evidence that movement–music synchrony may be particularly important for optimizing frontal–parietal integration. The stronger effects of FE+TJ compared to FE+BDJ are consistent with recent findings that continuous, rhythmically coordinated movements produce greater neuroplastic changes than discrete exercises (Heijs et al., 2025).

The current FC patterns also differ from previous music-only interventions that primarily reported frontotemporal connectivity changes (Luo et al., 2025), suggesting that Five-Element Music may exert unique effects when paired with movement. This interpretation is supported by our finding that FE alone produced only modest FC increases (CH45-CH9 FC = 0.33), while TJ alone showed even weaker effects (CH45-CH9 FC = 0.12). The nonlinear enhancement in combined conditions supports the “dual pathway” model proposed by Kolodny et al. (2017), where auditory and motor inputs converge to optimize network efficiency. Our results extend this model by identifying specific channel pairs (CH45-CH9 and CH29-CH6) that appear particularly responsive to combined stimulation, potentially serving as biomarkers for effective interventions in aging populations. These findings complement existing evidence that mind–body practices improve brain structure and function (G. Li et al., 2024), while introducing new evidence that their benefits can be significantly amplified when carefully paired with culturally congruent auditory stimuli (Tao et al., 2016).

Limitations of this study: A few limitations of this study should be acknowledged. First, while this study assessed cognitive function through working memory tasks, using a broader range of cognitive assessments, such as the digit span reverse task, could have provided a more comprehensive measure of cognitive abilities. Second, the analyses did not account for potential demographic variables, such as gender or education level, which could have influenced the results. Third, the emotional regulation task used in this study may not have been fully standardized, potentially introducing variability in task performance. These factors should be considered in future research to enhance the robustness of the findings.

## 5. Conclusions

The combination of Five-Element Music and Taijiquan significantly improved emotion regulation, working memory, and functional brain connectivity in older adults. The FE+TJ intervention enhanced acceptance rates for high-intensity emotional stimuli, increased 2-back task accuracy, and strengthened prefrontal–parietal connectivity (CH45-CH9, CH29-CH6), indicating improved neural coordination. These findings highlight the synergistic effects of integrating auditory stimulation with mind–body exercise, offering a promising intervention for enhancing cognitive and emotional health in aging populations. Future research should explore long-term benefits and broader applications.

## Figures and Tables

**Table 1:** Demographic characteristics.

	Male (48%)	Female (52%)
Age	64.1 (3.2)	65.0 (4.2)
BMI (kg/m^2^)	24.35 (5.54)	24.30 (6.70)
Education		
<6 years	5.7%	19.5%
6–9 years	19.2%	29.5%
9–12 years	26.7%	32.2%
12–16 years	28.1%	17.7%
>16 years	20.3%	1.1%

Note: The data are presented in the form of mean (standard deviation).

**Table 2:** Differences in emotional regulation and working memory under different conditions.

	Baseline	FE	TJ	BDJ	FE+TJ	FE+BDJ	F	*p*	η²_P_
Emotional regulation task behavioral variables									
Acceptance use: overall (%)	57.41 (23.39)	63.33 (19.95)	58.37 (22.56)	56.85 (22.07)	67.24 (14.25)	59.23 (19.24)	1.096	0.364	0.026
Acceptance use: low intensity (%)	62.29 (22.15)	66.29 (16.23)	63.19 (21.26)	61.13 (21.56)	69.27 (12.72)	63.29 (14.77)	1.898	0.096	0.044
Acceptance use: high intensity (%)	52.54 (24.28)	60.37 (14.34)	53.54 (25.27)	52.58 (23.88)	65.21 (16.63)	55.16 (21.59)	4.659	0.001	0.102
N-back task behavioral variables									
0-back average accuracy	0.82 (0.29)	0.79 (0.33)	0.83 (0.31)	0.81 (0.30)	0.82 (0.26)	0.83 (0.31)	0.981	0.430	0.023
0-back average RT (ms)	909.36 (187.45)	923.66 (201.29)	924.12 (223.13)	911.12 (199.72)	920.31 (242.12)	917.14 (217.91)	0.330	0.894	0.008
2-back average accuracy	0.76 (0.21)	0.81 (0.09)	0.81 (0.11)	0.77 (0.15)	0.83 (0.10)	0.79 (0.23)	4.202	0.001	0.093
2-back average RT (ms)	1294.29 (185.35)	1162.13 (127.14)	1263.19 (192.19)	1271.56 (276.48)	1127.12 (146.29)	1270.34 (201.43)	5.354	0.001	0.115

Note: The data are presented in the form of mean (standard deviation). Abbreviation: FE: Five-Element Music; TJ: Taijiquan; BDJ: Baduanjin; FE+TJ: combined practice of Five-Element Music and Taijiquan; FE+BDJ: combined practice of Five-Element Music and Baduanjin.

**Table 3:** Significantly different FC values of channels under different conditions.

CH↔CH	FC Values						Condition		
	Baseline	FE	TJ	BDJ	FE+TJ	FE+BDJ	F	*p*	P_FDR_
CH 45↔CH 9	0.25 (0.11)	0.33 (0.15)	0.12 (0.05)	0.25 (0.11)	0.83 (0.23)	0.09 (0.07)	10.23	<0.001	0.008
CH 29↔CH 6	0.04 (0.03)	0.13 (0.07)	0.15 (0.06)	0.07 (0.04)	0.79 (0.17)	0.03 (0.02)	9.87	<0.001	0.012
CH 45↔CH 11	0.08 (0.05)	0.34 (0.08)	0.26 (0.07)	0.05 (0.04)	0.13 (0.09)	0.63 (0.21)	8.55	<0.001	0.015

Note: P_FDR_: *p*-values are adjusted using the false discovery rate (FDR) correction. Abbreviations: FC: functional connectivity; CH: channel.

## Data Availability

The original contributions presented in this study are included in the article. Further inquiries can be directed to the corresponding author.
